# Defining the ABC of gene essentiality in streptococci

**DOI:** 10.1186/s12864-017-3794-3

**Published:** 2017-05-31

**Authors:** Amelia R. L. Charbonneau, Oliver P. Forman, Amy K. Cain, Graham Newland, Carl Robinson, Mike Boursnell, Julian Parkhill, James A. Leigh, Duncan J. Maskell, Andrew S. Waller

**Affiliations:** 1 Animal Health Trust, Lanwades Park, Newmarket Suffolk, UK; 2 Department of Veterinary Medicine, University of Cambridge, Cambridge, UK; 3 The Wellcome Trust Sanger Institute, Wellcome Trust Genome Campus, Hinxton, UK; 4 Department of Chemistry and Biomolecular Sciences, Macquarie University, Sydney, Australia; 5 The School of Veterinary Medicine and Science, University of Nottingham, Sutton Bonington, Leicestershire Nottingham, UK

**Keywords:** Transposon, Sequencing, Essentiality, Barcode

## Abstract

**Background:**

Utilising next generation sequencing to interrogate saturated bacterial mutant libraries provides unprecedented information for the assignment of genome-wide gene essentiality. Exposure of saturated mutant libraries to specific conditions and subsequent sequencing can be exploited to uncover gene essentiality relevant to the condition. Here we present a barcoded transposon directed insertion-site sequencing (TraDIS) system to define an essential gene list for *Streptococcus equi* subsp. *equi*, the causative agent of strangles in horses, for the first time. The gene essentiality data for this group C *Streptococcus* was compared to that of group A and B streptococci.

**Results:**

Six barcoded variants of pGh9:IS*S1* were designed and used to generate mutant libraries containing between 33,000-66,000 unique mutants. TraDIS was performed on DNA extracted from each library and data were analysed separately and as a combined master pool. Gene essentiality determined that 19.5% of the *S. equi* genome was essential. Gene essentialities were compared to those of group A and group B streptococci, identifying concordances of 90.2% and 89.4%, respectively and an overall concordance of 83.7% between the three species.

**Conclusions:**

The use of barcoded pGh9:IS*S1* to generate mutant libraries provides a highly useful tool for the assignment of gene function in *S. equi* and other streptococci. The shared essential gene set of group A, B and C streptococci provides further evidence of the close genetic relationships between these important pathogenic bacteria. Therefore, the ABC of gene essentiality reported here provides a solid foundation towards reporting the functional genome of streptococci.

**Electronic supplementary material:**

The online version of this article (doi:10.1186/s12864-017-3794-3) contains supplementary material, which is available to authorized users.

## Background

Strangles, caused by *Streptococcus equi* subspecies *equi* (*S. equi*), is one of the most frequently diagnosed infectious equine diseases worldwide. *S. equi* is a Gram positive bacterium belonging to the Lancefield group C family of streptococci [1]. *S. equi* is closely related to the group A *Streptococcus*, *Streptococcus pyogenes* (*S. pyogenes*) [2] and the group B *Streptococcus*, *Streptococcus agalactiae* (*S. agalactiae*) [3], both of which are important human pathogens. *S. pyogenes* causes impetigo, pharyngitis, scarlet fever and necrotising fasciitis [4–6] and *S. agalactiae* causes meningitis, pneumonia and sepsis in neonates [7], in addition to mastitis in cattle [8] and streptococcosis in fish [9].

The increased accessibility of next-generation sequencing (NGS) technologies has facilitated the development of a variety of transposon-genome junction sequencing techniques, which combine dense mutant libraries and sequencing to identify essential bacterial genomes and assign gene function. The precise details of these methods: TraDIS, Tn-seq, HITS, INSeq and PIMMS vary from one another [10–14], yet all produce similar end-point data [15]. Each technique employs a transposon delivery vector to produce a library of random transposition mutants within the bacterial genome. Viable mutants contain transposons that have inserted into non-essential genes, with insertions into essential genes proving lethal. NGS of transposon-genome junctions in saturated transposon mutant libraries permits the simultaneous identification of potentially hundreds of thousands of unique insertion sites, providing data pertaining to gene essentiality at the most basic level. Exposure of mutant libraries to varying experimental conditions, however, enables the relative fitness and conditional essentiality of each gene to be determined. In recent years, a range of essential bacterial genomes have been published using transposon directed sequencing methods [10, 11, 14, 16–21]. Interrogating genomes in this way provides an unprecedented insight into genome-wide fitness, especially when libraries are subjected to disease relevant conditions either in vitro [10, 16, 22] or in vivo [23–27].

Here, we present a transposon directed insertion-site sequencing (TraDIS) system which is conducted using standard Illumina sequencer protocols. Dense mutant libraries utilising the plasmid pGh9 carrying the insertion element IS*S1* (pGh9:IS*S1*) [28], have previously been utilised with success in *Streptococcus uberis* (*S. uberis*) [14]. We modified pGh9:IS*S1* within the 5’ terminal of IS*S1* to create six barcoded transposons. The six independent libraries were generated and sequenced after growth in rich media. Data for each library was compared and combined, providing a blue-print data set for the subsequent analysis of conditional fitness and gene essentiality assignment in *S. equi*. The agreement of gene essentiality between our *S. equi* TraDIS data and Tn-Seq data from the close relatives *S. pyogenes* and *S. agalactiae* was determined. KEGG (Kyoto encyclopaedia of genes and genomes) pathways were attributed to the essential gene sets of *S. equi*, *S. pyogenes* and *S. agalactiae* to unveil the key biochemical pathways in which they are involved.

## Methods

### Barcoding IS*S1*

Five barcoded variants of the plasmid pGhost9:IS*S1* [28] were generated using the primers listed in (Additional file 1: Table S1) to mutate the two bases (CA) located three and four bases downstream of the IS*S1* inverted repeat (Additional file 1: Figure S1 for plasmid map and Additional file 1: Figure S2 for PCR design). The new plasmids: pGh9:ISS*1*:TC, pGh9:IS*S1*:AG, pGh9:IS*S1*:AC, pGh9:IS*S1*:CT and pGh9:IS*S1*:GA contained the alternative bases TC, AG, AC, CT or GA, respectively at these positions. For clarity, the original pGhost9:IS*S1* will be referred to as pGh9:IS*S1*:CA in this manuscript with final libraries referred to as CA, TC, AG, AC, CT and GA. The methodology utilised for this process is provided in Additional file 1.

### Generation of transposon libraries

*S. equi* strain 4047 (*Se*4047) cells were transformed with the desired pGh9:IS*S1* plasmid by electroporation as previously described [29]. Transformants were grown for 3 h in Todd-Hewitt broth (THB) at 28 °C, permitting extrachromosomal plasmid replication. Transformants were grown on Todd-Hewitt agar (THA) supplemented with 0.5 μg/ml erythromycin (THAE) for 3 days at 28 °C. A colony of transformants was then grown overnight at 28 °C in THB supplemented with 0.5 μg/ml erythromycin (THBE). Overnight cultures were heat shocked at 40 °C for 3 h resulting in random transposition of IS*S1* and the plasmid into the bacterial chromosome. Transposition frequency was determined by counting the colony forming units per millilitre of transposants on THAE versus THA. Transposants were selected by overnight growth at 37 °C in a humidified atmosphere containing 5% CO_2_ on 30 large (150 mm diameter) THAE plates supplemented with 0.03 μg/ml of hyaluronidase. Pools of random transposon mutants (transposon libraries) were harvested from the plates by washing with THB containing 25% glycerol and the bacterial suspension stored at -20 °C. The transposon libraries were then grown at 37 °C in a humidified atmosphere containing 5% CO_2_ to an OD_600nm_ of 0.3 in THBE, 2.5 ml of the culture was centrifuged at 10,000 xg for 5 min and the bacterial pellet stored at -20 °C.

### Effect of barcoded IS*S1* on library growth

Each of the six barcoded libraries were grown overnight in THBE at 37 °C in a humidified atmosphere containing 5% CO_2_ alongside wild-type *Se*4047, which was grown in THB. Cultures were diluted to an initial OD_600nm_ of approximately 0.08 and incubated under the same conditions. The OD_600nm_ was measured every 30 min until stationary phase. The growth curves were completed in triplicate, with each replicate conducted on different days and from different stored aliquots. Doubling times were calculated from the mean exponential phase data for each library and *Se*4047. The mean doubling times of the libraries were tested for statistical significance using the Student’s *t*-test.

### Stability of integrated pGh9:IS*S1*

Ninety-five colonies recovered from library CA were grown overnight in THBE at 37 °C in a humidified atmosphere containing 5% CO_2_, before they were combined to generate P0. The 95 mutant pool was then passaged twice overnight under the same conditions to produce P1 and P2. 2.5 ml of each culture was centrifuged at 10,000 xg for 5 min and the bacterial pellet stored at -20 °C.

### DNA preparation and sequencing by TraDIS

DNA was extracted from the six barcoded mutant libraries and the three stability libraries cell pellets using a GenElute column kit according to the manufacturer’s instructions for Gram positive bacteria (Sigma-Aldrich). DNA was quantified using the Qubit dsDNA BR assay kit. 1.5 μg DNA was fragmented by sonication using a Misonix XL 2020 Ultrasonic Liquid Processor (cup horn arrangement) to produce fragments in the range of 200-800 bp, with 800 bp fragments being most prevalent. A Y-adaptor generated in-house using Illumina multiplexing adaptor sequences (Oligonucleotide sequences © 2007- 2012 Illumina, Inc. All rights reserved) was ligated to 1 μg of fragmented DNA using the NEBNext Ultra II DNA library prep kit for Illumina (New England Biolabs) according to the manufacturer's instructions for End Repair and Adaptor Ligation (see Additional file 1 for adaptor generation protocol). Fragments were purified using AMPure XP beads (Agencourt, Beckman Coulter) with a bead to DNA ratio of 1:1, according to manufacturer’s instructions.

Incubation of adaptor ligated DNA with the restriction enzyme *Sma*I for two hours at 25 °C was used to cleave the pGh9:IS*S1* plasmid 33 bp upstream of the sequence encoding IS*S1* in order to minimise the amount of TraDIS reads mapping to plasmid. AMPure XP beads with a bead to DNA ratio of 1.8:1 were used to clean up the digest reaction, according to manufacturer’s instructions. The amount of DNA recovered was quantified using the Qubit dsDNA HS assay kit. As recommended by Langridge et al. 100 ng of library DNA was PCR amplified for 20 cycles according to 1.4C of the NEBNext Ultra II DNA library prep kit protocol. Amplification utilised the specific IS*S1* primer and indexing PCR primer, which facilitated the attachment of the resultant product to the sequencing flow cell (Additional file 1: Table S1). The regions that were amplified span the 5' end of IS*S1* and the site of transposition in the *S. equi* genome. The use of a Y-adaptor enabled amplification of IS*S1* containing fragments only, as reverse amplification could not occur until the specific IS*S1* primer had amplified, generating a complementary Y-adaptor sequence for the indexing PCR primer to bind (see Additional file 2 for a figure illustrating PCR strategy).

AMPure XP beads with a bead to DNA ratio of 0.8:1 were used to remove small PCR products, non-ligated adaptors and primer dimers. The concentrations of the libraries were calculated using the Kapa Biosystems library quantification kit, with average fragment sizes estimated from gel electrophoresis. The amplified libraries were then single-end sequenced using the Illumina MiSeq, with the six barcoded libraries as two triplex runs and the stability libraries as one run in triplex. All libraries were loaded at 10 pM. The barcoded libraries were combined with 40% PhiX (Illumina) and the stability libraries combined with 90% PhiX to increase cluster diversity. For each run, 3.4 μl of the custom Read 1 primer (Additional file 1: Table S1) was added to the Read 1 primer mix of the MiSeq cartridge (Illumina) to enable sequencing of PhiX and to generate reads beginning with the barcoded IS*S1*. A custom Index Read primer (Additional file 1: Table S1) was also loaded into the MiSeq cartridge according to the manufacturer’s instructions. Fastq only files were generated according to the following settings; TruSeq LT, 1 index read, 76 cycles, adaptor trimming unchecked and custom indexing primer selected.

### Analysis of sequencing data

Raw demultiplexed fastq files were analysed using the Bio-TraDIS scripts made available by the Sanger Wellcome Trust Institute [30] (https://github.com/sanger-pathogens/Bio-Tradis). Initially, the single command pipeline script, bacteria_tradis, was utilised. The pipeline filtered and removed reads according to the transposon tag specified (e.g. CAGAAAACTTTGCAACAGAACC for library CA). After tag removal, the remaining 46 bp of *S. equi* DNA were mapped to the *Se*4047 reference genome using SMALT short read mapper, producing a plot file of insertion sites for viewing in the Artemis genome browser [31], and for downstream analysis. The default transposon tag mismatch of 0 was maintained, however a mapping threshold of 100% was set (SMALT parameter y = 1) to improve accuracy and confidence in the assignment of insertion sites. Next, the plot files generated from bacteria_tradis were analysed by tradis_gene_insert_sites, generating a readable document of unique insertion sites, total read counts and insertion indices, per gene. Unique insertion sites represented by two or fewer reads were not included in the analysis. The output file from tradis_gene_insert_sites was then used in tradis_essentiality to determine the essential genome of *S. equi*. Tradis_essentiality uses the empirically observed bimodal distribution of the insertion indices (essential and non-essential peaks) to fit gamma distributions. Log_2_ likelihood ratios (LLR) are calculated between the gamma distributions, with genes assigned a LLR of less than -2 identified as essential, more than 2 as non-essential and between the two values as ambiguous [30]. Essential and ambiguous changepoints were calculated from these LLRs to categorise genes into essential, ambiguous and non-essential groups. Essentialities of genes with multiple genomic copies were called as ‘not defined’ due to reduced confidence in read mapping. The fastq files from each library were combined, clipped of their first 2 bp to standardise the IS*S1* tag at the beginning of each read and re-analysed to generate a master library, from which final gene essentiality is reported in this study. To identify any insertion site bias, master library mapped reads, with duplicates removed, were parsed through WebLogo, to determine the probability of each nucleotide occurring at positions 1-20 (the insertion site to 20 bp downstream) [32].

### Comparative analysis of *S. equi* TraDIS to *S. pyogenes* and *S. agalactiae* Tn-Seq data

Gene essentiality calls of *S. pyogenes* strain M1T1 5448 and *S. agalactiae* strain A909 were retrieved from the supplementary information provided by Le Breton et al. and Hooven et al [19, 20]. In these studies, each gene of *S. pyogenes* and *S. agalactiae* was reported as essential, critical, non-essential or not defined/non-conclusive. KEGG pathway enrichment was completed on the essential and critical genes of *S. pyogenes* and *S. agalactiae* in addition to the essential and ambiguous genes of *S. equi*, using the gene set enrichment analysis available as an online tool on Genome 2D (http://pepper.molgenrug.nl/index.php/gsea-pro) [33]. The KEGG pathways attributed to the essential, critical and ambiguous genes were compared between the three bacteria. Gene orthologues were also identified between *S*e4047 and *S. pyogenes* strain MGAS5005 (reference strain used by Le Breton et al. for M1T1 5448)*, S*e4047 and *S. agalactiae* strain A909 and between *S. pyogenes* strain MGAS5005 and *S. agalactiae* strain A909 using the online tool OrtholugeDB (http://www.pathogenomics.sfu.ca/ortholugedb/) [34]. The essentiality calls of each orthologous gene pair were compared to determine concordance. All results generated from OrtholugeDB were included in the analysis, except for duplicated calls where multiple copies of a gene exist in either bacterium or when gene essentiality is not defined or non-conclusive.

## Results and discussion

### Insertion of barcoded pGh9:IS*S1* is random, stable and dense in *S. equi*

**Fig. 1:**
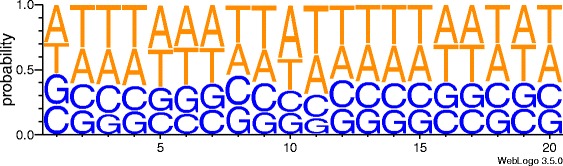
WebLogo of IS*S1* insertion sites in *S. equi*. Data from six barcoded IS*S1* mutant libraries in *S. equi* were combined to generate a master library. Unique sequence reads were isolated from the master library data set and parsed through WebLogo [32] to identify any insertion site bias between the insertion site and 20 bp downstream. No insertion site bias was found

To generate our six *S. equi* mutant libraries, we utilised six barcoded pGh9:IS*S1* plasmids. There were no significant differences in the mean doubling time of *Se*4047 relative to those of the six barcoded libraries (*p* = 0.48) (see Additional file 1 for average growth curve plot). Transposition frequencies of between 3.5^-3^ and 7.8^-3^ were observed across the six barcoded libraries, which is comparable to the frequency of 4.9^-3^ reported by Magiun et al. where pGh9:IS*S1* was transposed into *Lactococcus lactis* (*L. lactis*) strain IL1403. The transposition frequency of pGh9:IS*S1* in *S. equi* was also comparable to that of the transposon, *Krmit*, in *S. pyogenes* (4^-3^) [14], but was higher than *Himar1*, a mini-transposon, in *S. agalactiae* (10^-4^–10^-6^) [13]. In common with previous studies that identifed IS*S1* transposition sites [14, 28], no specific sequence motif was observed at the transposition sites of IS*S1* in *S. equi* (Fig. 1). The probability of either an A or a T occuring at any position between the insertion site and 20 bp downstream, was between 54% to 70% per bp highlighting a modest preference of IS*S1* for AT rich regions, which is in agreement with the overall AT content of the *S. equi* genome (58.7%) [2].

To determine the stability of pGh9:IS*S1* transposition, 95 colonies from library CA were pooled (P0) and passaged twice. Sequencing of P0 identified 95 insertion sites, representing 84 genes. Ninety-five insertion sites were also identified in P1, in the same 84 genes, except that an additional mutant was identified in SEQ_1253 and a SEQ_0705 mutant was lost. For P2, 92 mutants were identified, representing 83 of the same genes. The SEQ_1253 mutant gained in P1 was lost, in addition to two other mutants in SEQ_1270 and SEQ_1697. The gain then loss of a mutant in SEQ_1253 is likely due to sample preparation/sequencing differences with the remaining losses due to fitness effects following transposition of IS*S1*. Our data support the stability of pGh9:IS*S1* in the *S. equi* genome and provide evidence that any onward translocation of pGh9:IS*S1* post-transposition occurs at an undetectable level.

Our technique for the generation of transposon libraries, in common with the PIMMS method utilised for the identification of IS*S1* insertion sites in *S. uberis* [14], does not attempt to eliminate the plasmid after transposition. IS*S1* duplicates on transposition generating a copy of pGh9, flanked on both sides by IS*S1*, resulting in the presence of undesirable IS*S1*-plasmid fragments in library DNA [28]. PIMMS employs an inverse PCR of re-circularised DNA fragments to identify genomic sequences flanking IS*S1* insertion sites [14]. Our TraDIS approach utilised Y-adapters to specifically amplify from IS*S1* generating both IS*S1*-plasmid and IS*S1*-genome fragments (see Additional file 2 for a figure illustrating PCR strategy). Incubation of Y-adaptor ligated DNA with *Sma*I before PCR cleaved IS*S1*-plasmid fragments, such that these undesirable sequence reads accounted for only 5 to 10% of the final dataset. Thirteen *Sma*I restriction sites are present in the *Se*4047 genome and it is predicted that sequence reads mapping to the immediate regions surrounding these sites will similarly be lost from the final TraDIS data set. An alternative restriction enzyme may be preferred for use in other bacteria. A list of restriction enzymes that cut within 200 bp of the 5’ end of IS*S1* is provided in (Additional file 1: Table S2).

The fastq files from each barcoded library were combined and reanalysed to generate a master library (Table 1). The master library represents sequencing data from two MiSeq runs, from which 37.6 million reads were obtained. Reads that contained the desired IS*S1* tag totalled 32.6 million of which 17.2 million (53%) mapped with 100% identity to the *Se*4047 genome. IS*S1*-plasmid reads account for some of the unmapped reads, however the majority are likely attributable to insufficient mapping quality using the high stringency criteria described above or through mapping to repetitive sequences within the *S. equi* genome [2].

**Table 1:** Summary of TraDIS data obtained from sequencing six barcoded *S. equi* mutant libraries, generated with pGh9:IS*S1*

Library	Unique insertion sites in genes	Total reads in genes	Genes containing insertions (% of total genes)	Library saturation (insertion every n bp in genes)
CA	54,815	1,645,725	1,787 (87.6)	35
TC	51,827	2,162,710	1,804 (88.5)	37
AG	66,384	1,816,701	1,792 (87.9)	29
AC	35,592	3,290,822	1,797 (88.1)	54
CT	32,502	3,171,602	1,804 (88.5)	59
GA	44,761	2,650,678	1,815 (89)	43
master	208,531	14,825,797	1,935 (94.9)	9

Data from the six libraries were combined to generate the master library

On average, the master library contained an insertion every 9 bp in genes, representing a 79% increase in saturation when compared to insertions in the individual barcoded libraries. This considerable increase in library saturation did not greatly increase the number of genes represented in the master library, which was an average of 6.6% more than was found in the individual barcoded libraries. Our data demonstrate that IS*S1* transposition occurred reproducibly across the *S. equi* genome regardless of the barcoded IS*S1* that was used.

**Fig. 2:**
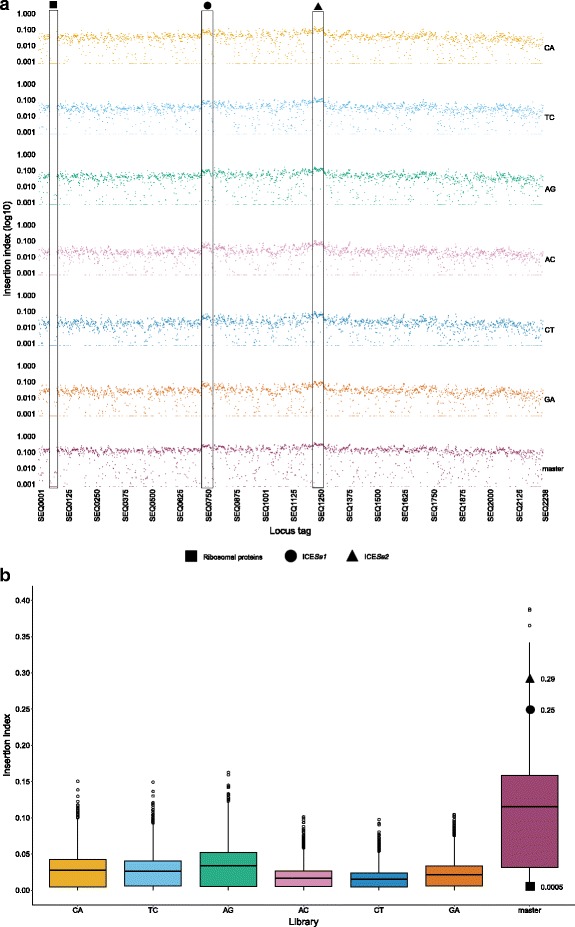
Insertion indices of *S. equi* genes disrupted by barcoded pGh9:IS*S1*. **a**. Insertion indices (log10) per gene is replicable between the six barcoded libraries. Each library is identified by its barcode on the right of the figure. The data was combined to generate a master library. Common peaks and troughs are evident; a decreased insertion index is clear in all libraries in a region of ribosomal proteins, with peaks in the integrative conjugative elements ICE*Se1* and ICE*Se2* visible. **b**. Box and whisker plot of the insertion indices of each barcoded library and the master library. The pooling of data to generate the master library was supported by the increased interquartile range and the elevated lower quartile range, increasing the robustness of the data set from which gene essentiality was determined. Average insertion indices from master library data in a region of ribosomal proteins, ICE*Se1* and ICE*Se2* are shown

The widespread distribution of IS*S1* transposition is evident from Fig. 2a, which shows common regions of increased and decreased transposition (insertion index (number of unique insertions/size of the gene)) across the six libraries. A low insertion index was observed in genes encoding ribosomal proteins, with increased insertion indices evident in regions of low GC content for example in the integrative conjugative element (ICE) *Se1* (ICE*Se1*) and ICE*Se2* (Fig 2a). The pooling of data to generate the master library was supported by the increased interquartile range observed in Fig. 2b. Pooling the data elevated the lower quartile range increasing the robustness of the data set from which gene essentiality was determined.

### The essential genome of *S. equi* is comparable to that of group A and B streptococci

**Fig. 3:**
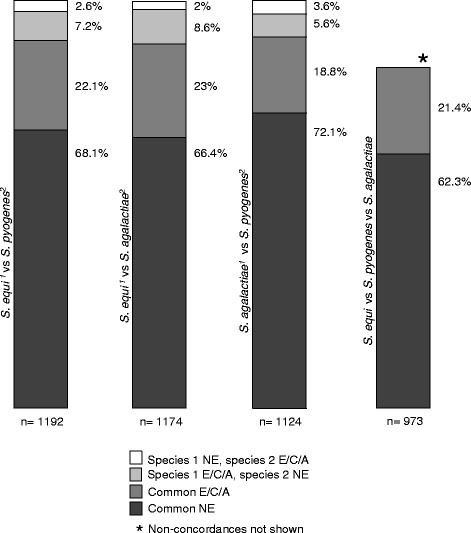
Gene essentiality concordance between a Group A, B and C streptococci. Essentiality between orthologous gene pairs in *S. equi*, *S. pyogenes* and *S. agalactiae* were compared. Orthologues were classified as either essential/critical/ambiguous concordant (E/C/A) or non-essential (NE) concordant. Non-concordances are also shown for 2-species comparisons only

Analysis of the master library with the tradis_essentiality TraDIS toolkit script [30] identified essential, ambiguous and non-essential genes based on the insertion index attributed to each gene. The tradis_essentiality script calculates the essential and ambiguous changepoints, from which gene essentiality is categorised. For the master data set, the essential and ambiguous changepoints were 0.0314 and 0.0408, respectively. Diagnostic plots and the results files generated by tradis_essentiality are available in Additional files 3 and 4. Using these thresholds, 19.5% of the *Se*4047 genome was found to be essential, 1.2% ambiguous, 73.4% non-essential and 5.8% not defined. The proportion of essential genes in *Se*4047 is similar to the 12% and 13.5% essential genes in *S. pyogenes* [14] and *S. agalactiae* [13], respectively. The essential gene sets for *Se*4047 were compared to those reported for *S. pyogenes* M1T1 5448 [20] and *S. agalactiae* A909 [19] (Additional file 5). There was 90.2% concordance of gene essentiality between *S. equi* and *S. pyogenes*; 89.4% between *S. equi* and *S. agalactiae*; 90.9% between *S. pyogenes* and *S. agalactiae* and 83.7% between the three species (Fig. 3). Our data highlight the similarities of the functional genomes of these different pathogens in support of previous studies that identified shared core and accessory genomes [2, 3]. In each species, libraries were generated using different transposons, prepared and analysed in different ways and yet identified common essential gene sets. Our data illustrate the compatibility of these methodologies and the reproducibility of essentiality assignments across these streptococci.

**Fig. 4:**
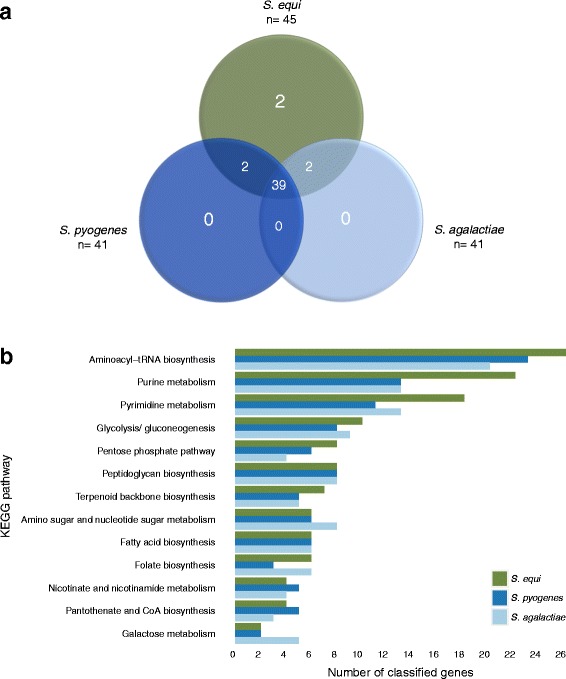
KEGG analysis of the essential/critical/ambiguous genes of Group A, B and C streptococci. **a**. Venn diagram showing the comparison of the KEGG categories assigned to the essential/critical/ambiguous genes of *S. equi, S. pyogenes* and *S. agalactiae*. The overlap of genes concludes that the essential pathways employed by the three different species are conserved. **b**. Barchart of the calls within most highly ranked KEGG pathways. The top KEGG categories in each species were consistent with one another

The biosynthetic pathways attributed to each species’ essential/critical/ambiguous gene set were identified by KEGG pathway analysis. Our analysis revealed that the essential/critical/ambiguous genes of *S. equi*, *S. pyogenes* and *S. agalactiae* were attributed to 45, 41 and 41 KEGG categories, respectively, 39 of which were shared between the three species (Fig. 4a) (Additional file 6). The 10 most prevalent essential/critical/ambiguous KEGG pathways in each species were compared (Fig. 4b). The highest-ranked categories were involved in key cellular processes such as aminoacyl-tRNA biosynthesis, purine and pyrimidine metabolism, glycolysis and gluconeogenesis, the pentose phosphate pathway and peptidoglycan biosynthesis. The top KEGG categories in each species were consistent with one another. However, the *S. equi* essential genome contained noticeably more genes implicated in purine and pyrimidine biosynthesis. This may reflect the larger essential gene set of *Se*4047 or may be attributed to the in vitro conditions in which our libraries were grown. A potential lack of purines and pyrimidines within our Todd Hewitt media could provide an alternative explanation for these findings. Interestingly, a broad transcriptional regulator *codY* [35], reported as non-essential in *S. agalactiae* [19] was found to be ambiguous and critical in *S. equi* and *S. pyogenes* [20], respectively.

### Novel features of the *S. equi* essential gene set

**Fig. 5:**
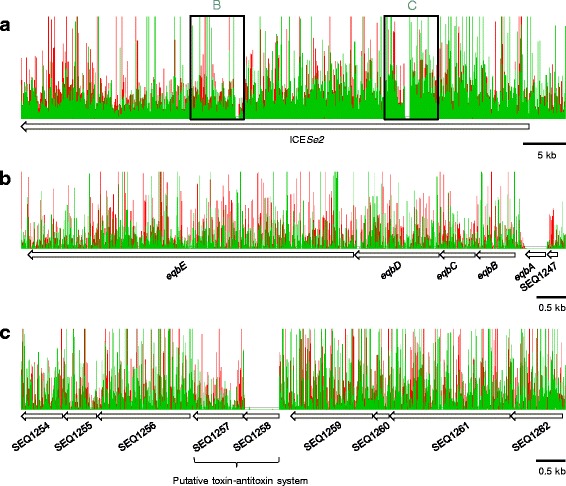
Sequence data from *S. equi* mutant libraries generated with IS*S1*. **a**. Overview of the integrative conjugative element, ICE*Se2*. Green and red peaks indicate reads mapping on the forward and reverse strand, respectively. IS*S1* insertion is dense in the region, except in two distinct genes, *eqbA* and SEQ_1258. The labels indicate the areas zoomed into in B and C of the figure. **b**. *eqbE* to SEQ_1247. IS*S1* insertion is dense, except for in *eqbA*, the regulator of the equibactin locus. Equibactin aids the acquisition of iron, which if unregulated leads to excessive iron import and a slow growth phenotype. **c**. SEQ_1254 to SEQ_1262. IS*S1* insertion is dense, except for in SEQ_1258, a putative antitoxin. ICE*Se2* encodes a putative toxin-antitoxin system which maintains the ICE in the bacterial genome. Both *eqbA* and SEQ_1258 were identifed as essential genes. Data is viewed in window size 9 for (**a**) and 3 for (**b**) and (**c**), with a maximum display value of 100 reads for ease of viewing. Data is viewed in Artemis [31]

Although the majority of essential genes in *S. equi* were similarly important in *S. pyogenes* and *S. agalactiae*, our analysis also identified some essential genes that were restricted to *Se*4047. *S. equi* produces a secreted molecule provisionally named equibactin, which aids the acquisition of iron in vitro [36] and is required for the full virulence of *S. equi* in a susceptible natural host [37]. Equibactin is synthesised by a non-ribosomal peptide synthesis system encoded in an operon (*eqbB* to *eqbN*) on the integrative conjugative element ICE*Se2* (Fig. 5a), which is regulated by the iron-dependent transcriptional repressor, EqbA [2, 36]. None of the genes *eqbB* to *N* were identified as essential in *S. equi*, in agreement with the free availability of iron in Todd-Hewitt media [29]. However, *eqbA* was essential for growth in vitro (Fig. 5b). Our results concur with those of Heather et al. who found that deletion of *eqbA* led to a slow-growth phenotype that was caused by excessive import of iron following de-regulation of the equibactin operon [36].

ICE*Se2* also contained a second essential gene, SEQ_1258 (Fig 3b). SEQ_1258 and SEQ_1257 are predicted to encode a novel toxin-antitoxin system in *S. equi* [2]. Toxin-antitoxin systems comprise a stable toxin and a labile antitoxin, which promote the maintenance of the element on which they are encoded within the bacterial genome [38]. Our data suggest that SEQ_1258 encodes the antitoxin in this system (Fig. 5c). The gene encoding the MosA antitoxin of the integrative conjugative element, SXT, of *Vibrio cholerae* was found to be essential, while *mosT*, encoding the toxin component could be deleted [38]. Recircularised extra-chromasomal copies of ICE*Se2* could not be recovered from *Se*4047 [36]. One possible explanation for this finding is that recircularisation of ICE*Se2* halts the production of the labile antitoxin, which cannot then neutralise the stable toxin still present in the cell. *S. equi* and *Streptococcus zooepidemicus* (*S. zooepidemicus*) share over 97% genetic identity [2], yet ICE*Se2* is not present in any strains of *S. zooepidemicus* studied to date [36, 39]. The maintenance of ICE*Se2* by its toxin-antitoxin system may restrict it to *S. equi*. Interestingly, decay of the equibactin locus in some *S. equi* isolates that were recovered from persistently infected horses did not include decay in SEQ_1258 [30], in agreement with the importance of the antitoxin as measured by TraDIS.

## Conclusions

We have successfully customised a barcoded TraDIS method based on the original method developed by Langridge et al. [10]. The barcoded TraDIS technique described herein is easily transferrable between laboratories and is conducted using accessible Illumina sequencer protocols, without the need for software alteration. Our barcoded technique will be of value to other researchers as it could be easily applied to other transposon systems for the study of a wide range of pathogenic bacteria. TraDIS and other transposon directed methods, represent a major advance in the study of gene function in bacteria. Utilising dense mutant libraries yields significant time and cost savings over the generation of traditional knockout strains, not only due to the speed at which saturated libraries can be generated, but also due to the ability to simultaneously identify conditionally essential genes. The use of barcoded pGh9:IS*S1* plasmids to generate mutant libraries of *S. equi* has provided a highly useful tool for the progression of TraDIS studies in this important bacterium. In particular, the ability to combine barcoded mutant libraries, challenge animals and then deconvolute the data generated has the potential to minimise the effects of animal to animal variation, enhance data quality and reduce the total number of animals required in future studies in accordance with the principles of the 3Rs; replacement, reduction and refinement [40]. Data from such in vivo studies, which are possible in the natural host, will provide an unprecedented insight into the pathways that underpin the virulence of *S. equi*, which will help to direct future vaccine research.

The shared essential gene set of group A, B and C streptococci provides further evidence of the close relationships of these important pathogenic bacteria. Our data suggest that the determination of gene essentiality for *S. equi* in the natural equine host is likely to also shed light on pathways of importance to the virulence of other streptococci. Therefore, this ABC of essential genes provides a solid foundation upon which to begin the process of reading the functional genomes of streptococci.

## Additional files

Additional file 1: Supplementary information: pGh9:IS*S1* plasmid map, method of barcoding IS*S1*, Y-adaptor generation, alternative restriction enzymes for plasmid depletion and effect of barcoded IS*S1* on library growth. (DOCX 71 kb)

Additional file 2: PCR strategy. 1. Adaptor ligated DNA. Y-adaptors were ligated onto DNA fragments containing either the desired IS*S1*-*S. equi* genome junction, IS*S1*- plasmid (pGh9) junction, only *S. equi* genome or only pGh9 DNA. 2. *Sma*I digestion of adaptor ligated DNA. Undesirable IS*S1*-plasmid junction containing DNA is depleted by digesting all fragments with the restriction enzyme, *Sma*I. This enzyme cuts pGh9 at a restriction site 33 bp from the IS*S1*-plasmid junction, which is rare in the *S. equi* genome. 3. PCR of digested DNA. 3.1. PCR phase 1. A specific IS*S1* forward primer was designed to amplify from the 5’ end IS*S1,* enriching for fragments containing an IS*S1* junction. Initial amplification with the specific IS*S1* primer generates an amplicon with a complementary adaptor sequence (shown in light blue). 3.2. PCR phase 2. The indexing PCR primer can now amplify from the complimentary adaptor sequence in the amplicon generated by phase 1. After phase 2, both primers can simultaneously amplify the amplicon. This strategy ensures that no reverse indexing primer amplification can occur until the forward primer has specifically amplified from IS*S1*. (PPTX 140 kb)

Additional file 3: Diagnostic plots: Gamma fit plots of each library and the Master data set produced by tradis_essentiality. The essential and ambiguous changepoints calculated by tradis_essentiality are shown on each graph. (PPTX 376 kb)

Additional file 4: *S. equi* gene essentialities: tradis_essentiality produces three separate files per library of either essential, ambiguous or all genes. Genes within the essential and ambiguous output files were identified in the all genes file and labelled as such, with the remainder identified as non-essential or not-defined. Each tab contains; locus tag, gene name, ncrna, start, end, strand, read count, insertion index, gene length, insertion count, function and essentiality. tab 1, CA library; tab 2, TC library; tab 3, AG library; tab 4, AC library; tab 5, CT library; tab 6, GA library; tab 7, Master library. (XLSX 1242 kb)

Additional file 5: Orthologous gene pair analysis: Gene essentialities from orthologous gene pair analysis between *S. equi*, *S. pyogenes* and *S. agalactiae*. tab 1, gene essentialities of orthologous gene pairs in *S. equi* and *S. pyogenes*; tab 2, gene essentialities of orthologous gene pairs in *S. equi* and *S. agalactiae*; tab 3, gene essentialities of orthologous gene pairs in *S. pyogenes* and *S. agalactiae*; tab 4, gene essentialities of orthologous gene pairs in *S. equi*, *S. pyogenes* and *S. agalactiae*. Each tab contains the locus tag and the corresponding gene essentiality. Orthologous genes are presented in the same rows. (XLSX 178 kb)

Additional file 6: KEGG analysis calls: Number of calls in each KEGG category assigned to the essential/critical/ambiguous genes of *S. equi*, *S. pyogenes* and *S. agalactiae*. Each KEGG category identified per species is presented, with the total number of calls within each category. (XLSX 13 kb)

## Abbreviations

3Rs: replacement, reduction and refinement
ICE: Integrative conjugative element
KEGG: Kyoto encyclopaedia of genes and genomes
NGS: Next-generation sequencing
THA: Todd-hewitt agar
THAE: Todd-hewitt agar with erythromycin
THB: Todd hewitt broth
THBE: Todd-hewitt broth with erythromycin
Tn-seq: Sequencing of transposon-genome junctions
TraDIS: Transposon directed-insertion site sequencing
