# Supplementary material for: Defining the ABC of gene essentiality in streptococci
Source: BMC Genomics. 2017 May 31;18:426. doi: 10.1186/s12864-017-3794-3 (PMC5452409; doi:10.1186/s12864-017-3794-3)
Supplement: Supplementary file 1 — Supplementary information: pGh9:ISS1 plasmid map, method of barcoding ISS1, Y-adaptor generation, alternative restriction enzymes for plasmid depletion and effect of barcoded ISS1 on library growth. (DOCX 71 kb) [file 12864_2017_3794_MOESM1_ESM.docx]

Additional file 1- supplementary information

**pGh9:IS*S1* plasmid map**

Fig S1. Barcoded pGh9:IS*S1* map. pGh9 is a temperature sensitive plasmid used for the delivery of IS*S1*. The IS*S1* transposase is flanked by an 18 bp inverted repeat and an 8 bp direct repeat. pGh9:IS*S1* contains an erythromycin resistance gene (*ermB*) used to select for successful transposition. The Gram-positive temperature sensitive replicase, RepA TS is utilised by pGh9. Restriction enzyme sites in close proximity to IS*S1* and its 2 bp barcode are shown.

**Barcoding IS*S1***

pGhost9:IS*S1* was digested with *Sal*I and *Sma*I to remove IS*S1*. The plasmid was treated with phosphatase to prevent re-ligation and was purified by gel extraction (Qiagen gel purification kit). Three Phusion PCRs were completed to generate each barcoded IS*S1*. In PCR 1, the forward primer (P1) amplified from the SmaI site within the plasmid, with the reverse primer (P3) spanning the site of base substitution, generating a 78 bp fragment (Fig. S2, Table S1).

Figure S2. Primer binding sites for the generation of barcoded IS*S1*. Primers P1 and P3 and primers P2 and P4 are utilised in two separate PCR reactions to generate two fragments of the new barcoded IS*S1*. A final PCR is conducted using the two products from the previous PCRs and primers P1 and P4 to generate the whole product. The red dots indicate the location of the two base pair barcode.

Table S1. Primer sequences

| Primer name | Sequence (5’-3’) | Concentration |
| --- | --- | --- |
| P1 | GACACGTCGACGGTATCGATAAGCTTG | 100 µM |
| P4 | GACACCCCGGGCTGCAGGAA | 100 µM |
| P2 TC* | GCAAAGTTTTCGAATAAGTCTATTTTAGTG | 100 µM |
| P3 TC* | CACTAAAATAGACTTATTCGAAAACTTTGC | 100 µM |
| P2 AG* | GCAAAGTTTTCCTATAAGTCTATTTTAGTG | 100 µM |
| P3 AG* | CACTAAAATAGACTTATAGGAAAACTTTGC | 100 µM |
| P2 AC* | GCAAAGTTTTCGTATAAGTCTATTTTAGTG | 100 µM |
| P3 AC* | CACTAAAATAGACTTATACGAAAACTTTGC | 100 µM |
| P2 CT* | GCAAAGTTTTCAGATAAGTCTATTTTAGTG | 100 µM |
| P3 CT* | CACTAAAATAGACTTATCTGAAAACTTTGC | 100 µM |
| P2 GA* | GCAAAGTTTTCTCATAAGTCTATTTTAGTG | 100 µM |
| P3 GA* | CACTAAAATAGACTTATGAGAAAACTTTGC | 100 µM |
| pGhost9:ISS1 forward | CTGGAACATCTGTGGTATGG | 100 µM |
| Adaptor primer 1Δ | P-GATCGGAAGAGCACACGTCT | 100 µM |
| Adaptor primer 2Δ | ACACTCTTTCCCTACACGACGCTCTTCCGATC×T | 100 µM |
| Specific IS*S1* primer | AATGATACGGCGACCACCGAGATCTACACGTTCATTGATATATCCTCGCTG | 25 µM |
| Indexing PCR primer 1** | CAAGCAGAAGACGGCATACGAGATCGGTTCGCCTTAACACTCTTTCCCTACACGACGCTCTTCCGATCT | 25 µM |
| Indexing PCR primer 2** | CAAGCAGAAGACGGCATACGAGATCGGTCTAGTACGACACTCTTTCCCTACACGACGCTCTTCCGATCT | 25 µM |
| Indexing PCR primer 4** | CAAGCAGAAGACGGCATACGAGATCGGTGCTCAGGAACACTCTTTCCCTACACGACGCTCTTCCGATCT | 25 µM |
| Custom read 1 primer | GTTCATTGATATATCCTCGCTGTCATTTTTATTCATTTTACACTAAAATAGACTTAT | 100 µM |
| Custom Index Read primer | AGATCGGAAGAGCGTCGTGTAGGGAAAGAGTGT | 100 µM |

* Barcode is underlined, ** Index sequence is underlined, × = Phosphorothioate bond, P = phosphorylation, Double underline = complementary sequence between adaptor primers, Δ = Oligonucleotide sequences © 2007- 2012 Illumina, Inc. All rights reserved.

PCR 2 utilised a forward primer also spanning the base substitution site (P2) and a reverse primer designed from the SalI site within the plasmid, generating an 848 bp fragment (Fig S2). Both PCR products were then used in PCR 3 which utilised the P1 and P4 primers, creating one product of 896 bp.

The PCR product was digested with *Sal*I, followed by *Sma*I as previously described and cloned back into the pGh9 plasmid. Plasmids were transformed into Escherichia coli TG1repA+ and grown at 37 ºC on Luria-Bertani agar containing erythromycin (150 µg/ml). pGh9:IS*S1* contains the erythromycin resistance gene, *ermB*, allowing selection of successfully cloned plasmids. Erythromycin resistant colonies were confirmed by PCR and were sequenced on an ABI3100 DNA sequencer with BigDye fluorescent terminators, using the pGhost9:IS*S1* forward primer, P1, P2, P3 and P4 in separate reactions.

**Y adaptor generation**

Fifteen µl of both adaptor primers 1 and 2 (Table S1) were combined and incubated at 95 ºC for 2 minutes, followed by an incremental decrease in temperature by 0.1 ºC to 20 ºC. The reactions were chilled on ice before 70 µl of ice cold MQ water was added to dilute the reaction to 15 µM.

**pGh9:IS*S1* DNA depletion**

Table S2. Restriction enzymes that cut within 200 bp from the 5’ end of IS*S1*. Restriction enzyme sites were identified using the online tool Restriction Mapper (http://www.restrictionmapper.org/).

| Name | Sequence | Overhang | Frequency | Cut Positions |
| --- | --- | --- | --- | --- |
| [*BsrBI*](http://rebase.neb.com/rebase/enz/BsrBI.html) | CCGCTC | blunt | 1 | 50 |
| [*MslI*](http://rebase.neb.com/rebase/enz/MslI.html) | CAYNNNNRTG | blunt | 1 | 62 |
| [*OliI*](http://rebase.neb.com/rebase/enz/OliI.html) | CACNNNNGTG | blunt | 1 | 62 |
| [*SmaI*](http://rebase.neb.com/rebase/enz/SmaI.html) | CCCGGG | blunt | 1 | 28 |
| [*XmnI*](http://rebase.neb.com/rebase/enz/XmnI.html) | GAANNNNTTC | blunt | 1 | 193 |
| [*AccI*](http://rebase.neb.com/rebase/enz/AccI.html) | GTMKAC | five_prime | 1 | 119 |
| [*AflIII*](http://rebase.neb.com/rebase/enz/AflIII.html) | ACRYGT | five_prime | 1 | 153 |
| [*AvaI*](http://rebase.neb.com/rebase/enz/AvaI.html) | CYCGRG | five_prime | 1 | 26 |
| [*AvaII*](http://rebase.neb.com/rebase/enz/AvaII.html) | GGWCC | five_prime | 1 | 86 |
| [*BbvI*](http://rebase.neb.com/rebase/enz/BbvI.html) | GCAGC | five_prime | 1 | 34 |
| [*BccI*](http://rebase.neb.com/rebase/enz/BccI.html) | CCATC | five_prime | 1 | 9 |
| [*CfrI*](http://rebase.neb.com/rebase/enz/CfrI.html) | YGGCCR | five_prime | 1 | 51 |
| [*DraII*](http://rebase.neb.com/rebase/enz/DraII.html) | RGGNCCY | five_prime | 1 | 86 |
| [*EcoRI*](http://rebase.neb.com/rebase/enz/EcoRI.html) | GAATTC | five_prime | 1 | 14 |
| [*NotI*](http://rebase.neb.com/rebase/enz/NotI.html) | GCGGCCGC | five_prime | 1 | 51 |
| [*PpuMI*](http://rebase.neb.com/rebase/enz/PpuMI.html) | RGGWCCY | five_prime | 1 | 86 |
| [*SpeI*](http://rebase.neb.com/rebase/enz/SpeI.html) | ACTAGT | five_prime | 1 | 38 |
| [*StyI*](http://rebase.neb.com/rebase/enz/StyI.html) | CCWWGG | five_prime | 1 | 100 |
| [*TseI*](http://rebase.neb.com/rebase/enz/TseI.html) | GCWGC | five_prime | 1 | 22 |
| [*BstXI*](http://rebase.neb.com/rebase/enz/BstXI.html) | CCANNNNNNTGG | three_prime | 1 | 64 |
| [*PstI*](http://rebase.neb.com/rebase/enz/PstI.html) | CTGCAG | three_prime | 1 | 24 |
| [*SacII*](http://rebase.neb.com/rebase/enz/SacII.html) | CCGCGG | three_prime | 1 | 63 |
| [*ApoI*](http://rebase.neb.com/rebase/enz/ApoI.html) | RAATTY | five_prime | 2 | 14, 167 |
| [*BamHI*](http://rebase.neb.com/rebase/enz/BamHI.html) | GGATCC | five_prime | 2 | 32, 68 |
| [*PleI*](http://rebase.neb.com/rebase/enz/PleI.html) | GAGTC | five_prime | 2 | 86, 175 |
| [*XbaI*](http://rebase.neb.com/rebase/enz/XbaI.html) | TCTAGA | five_prime | 2 | 44, 74 |
| [*XhoII*](http://rebase.neb.com/rebase/enz/XhoII.html) | RGATCY | five_prime | 2 | 32, 68 |
| [*TauI*](http://rebase.neb.com/rebase/enz/TauI.html) | GCSGC | three_prime | 2 | 53, 56 |

**Effect of barcoded IS*S1* on library growth**

Fig S3. Average growth curves of six *S. equi* mutant libraries generated with barcoded IS*S1*s. The libraries were grown in triplicate, alongside *Se*4047, the strain from which the libraries were made. Error bars were calculated from standard deviations.
