# Supplementary figures and images for: Defining the ABC of gene essentiality in streptococci
Source: BMC Genomics. 2017 May 31;18:426. doi: 10.1186/s12864-017-3794-3 (PMC5452409; doi:10.1186/s12864-017-3794-3)

## Slide 1
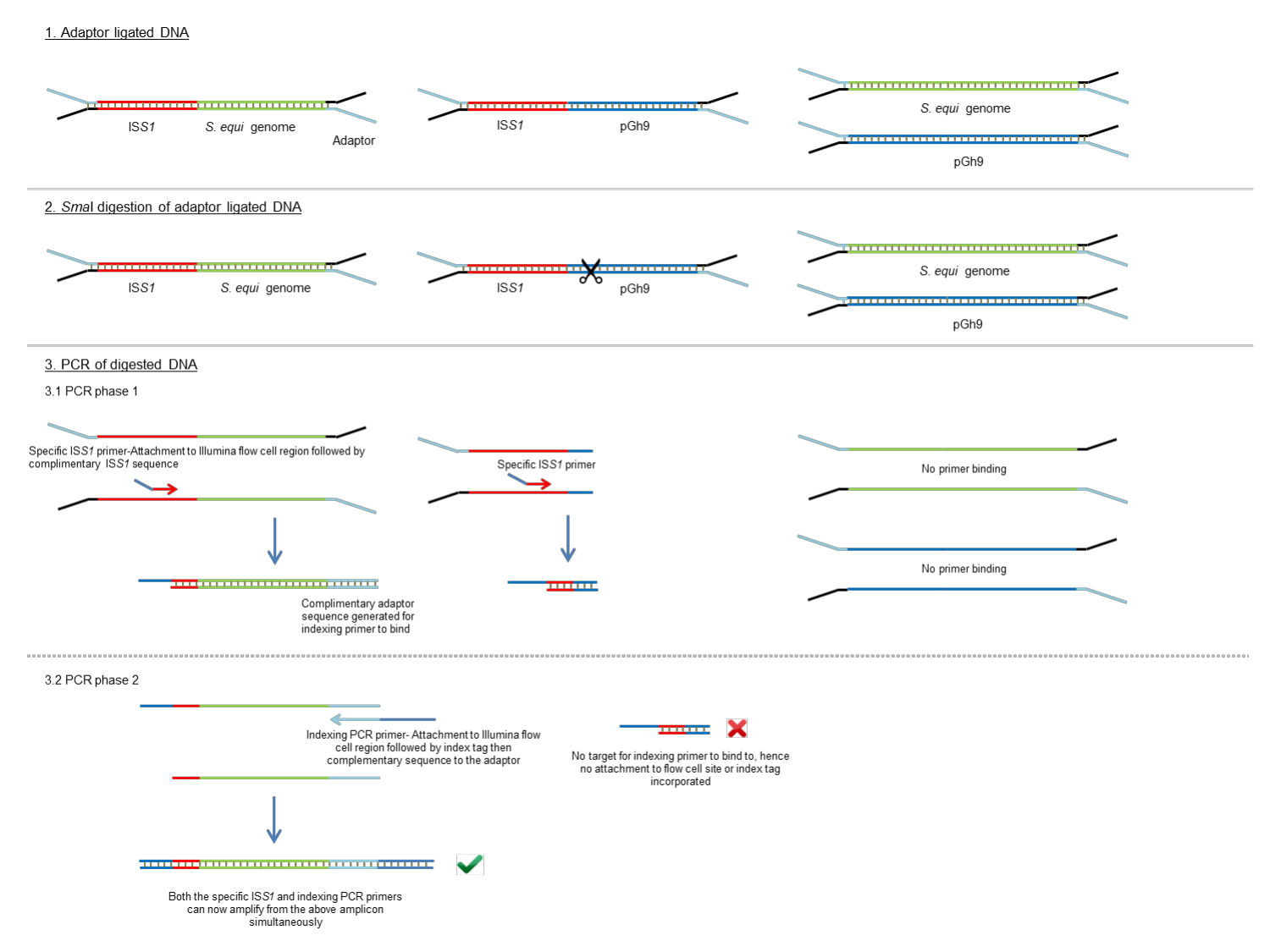

Supplement: Supplementary file 2 — PCR strategy. 1. Adaptor ligated DNA. Y-adaptors were ligated onto DNA fragments containing either the desired ISS1-S. equi genome junction, ISS1- plasmid (pGh9) junction, only S. equi genome or only pGh9 DNA. 2. SmaI digestion of adaptor ligated DNA. Undesirable ISS1-plasmid junction containing DNA is depleted by digesting all fragments with the restriction enzyme, SmaI. This enzyme cuts pGh9 at a restriction site 33 bp from the ISS1-plasmid junction, which is rare in the S. equi genome. 3. PCR of digested DNA. 3.1. PCR phase 1. A specific ISS1 forward primer was designed to amplify from the 5’ end ISS1, enriching for fragments containing an ISS1 junction. Initial amplification with the specific ISS1 primer generates an amplicon with a complementary adaptor sequence (shown in light blue). 3.2. PCR phase 2. The indexing PCR primer can now amplify from the complimentary adaptor sequence in the amplicon generated by phase 1. After phase 2, both primers can simultaneously amplify the amplicon. This strategy ensures that no reverse indexing primer amplification can occur until the forward primer has specifically amplified from ISS1. (PPTX 140 kb) [file 12864_2017_3794_MOESM2_ESM.pptx]
