# Supplementary material for: Defining the ABC of gene essentiality in streptococci
Source: BMC Genomics. 2017 May 31;18:426. doi: 10.1186/s12864-017-3794-3 (PMC5452409; doi:10.1186/s12864-017-3794-3)
Supplement: Supplementary file 3 — Diagnostic plots: Gamma fit plots of each library and the Master data set produced by tradis_essentiality. The essential and ambiguous changepoints calculated by tradis_essentiality are shown on each graph. (PPTX 376 kb) [file 12864_2017_3794_MOESM3_ESM.pptx]

## Slide 1
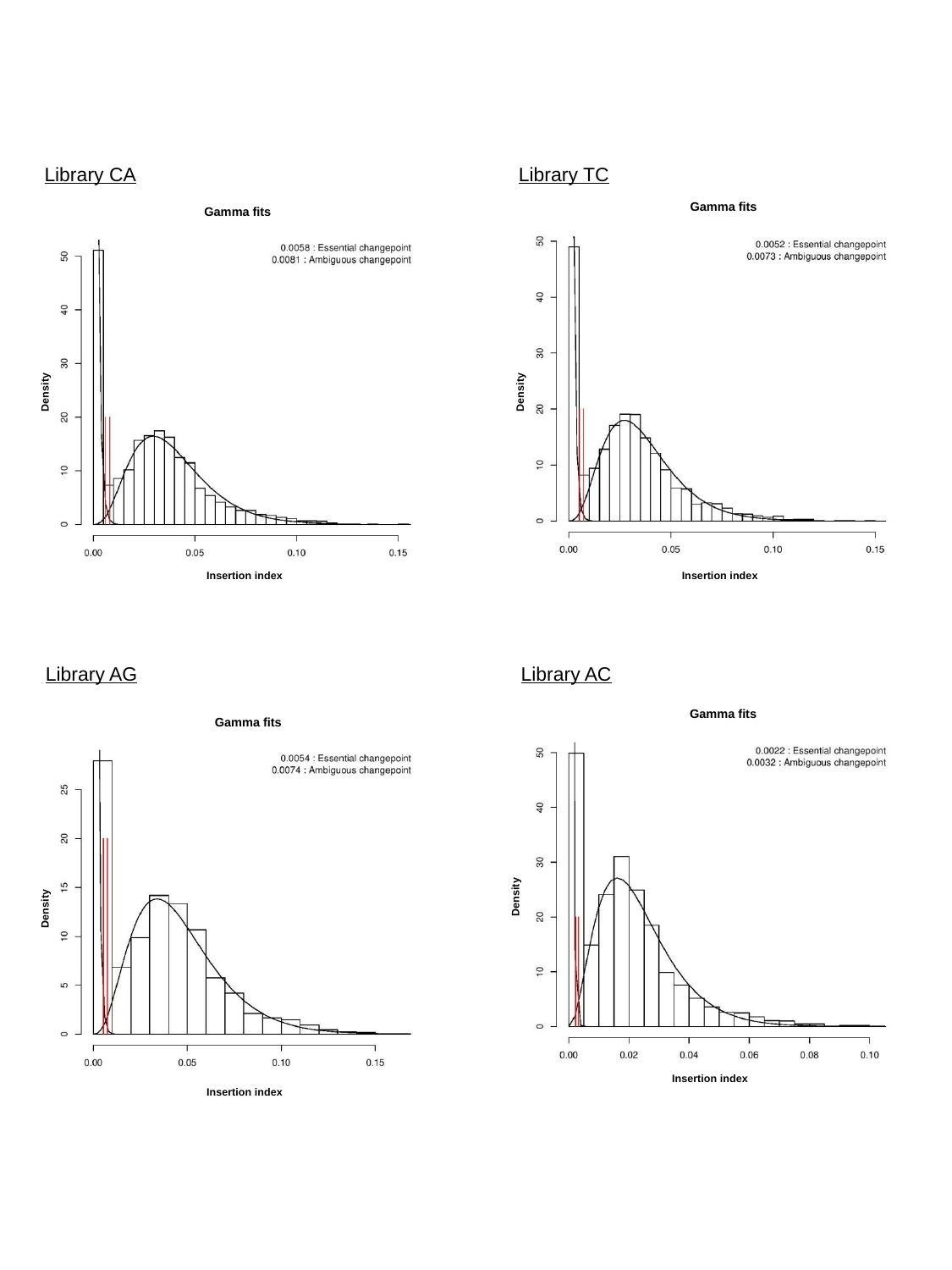

Library CA
Gamma fits
Density
Insertion index
Library TC
Gamma fits
Density
Insertion index
Library AG
Gamma fits
Density
Insertion index
Library AC
Gamma fits
Density
Insertion index

## Slide 2
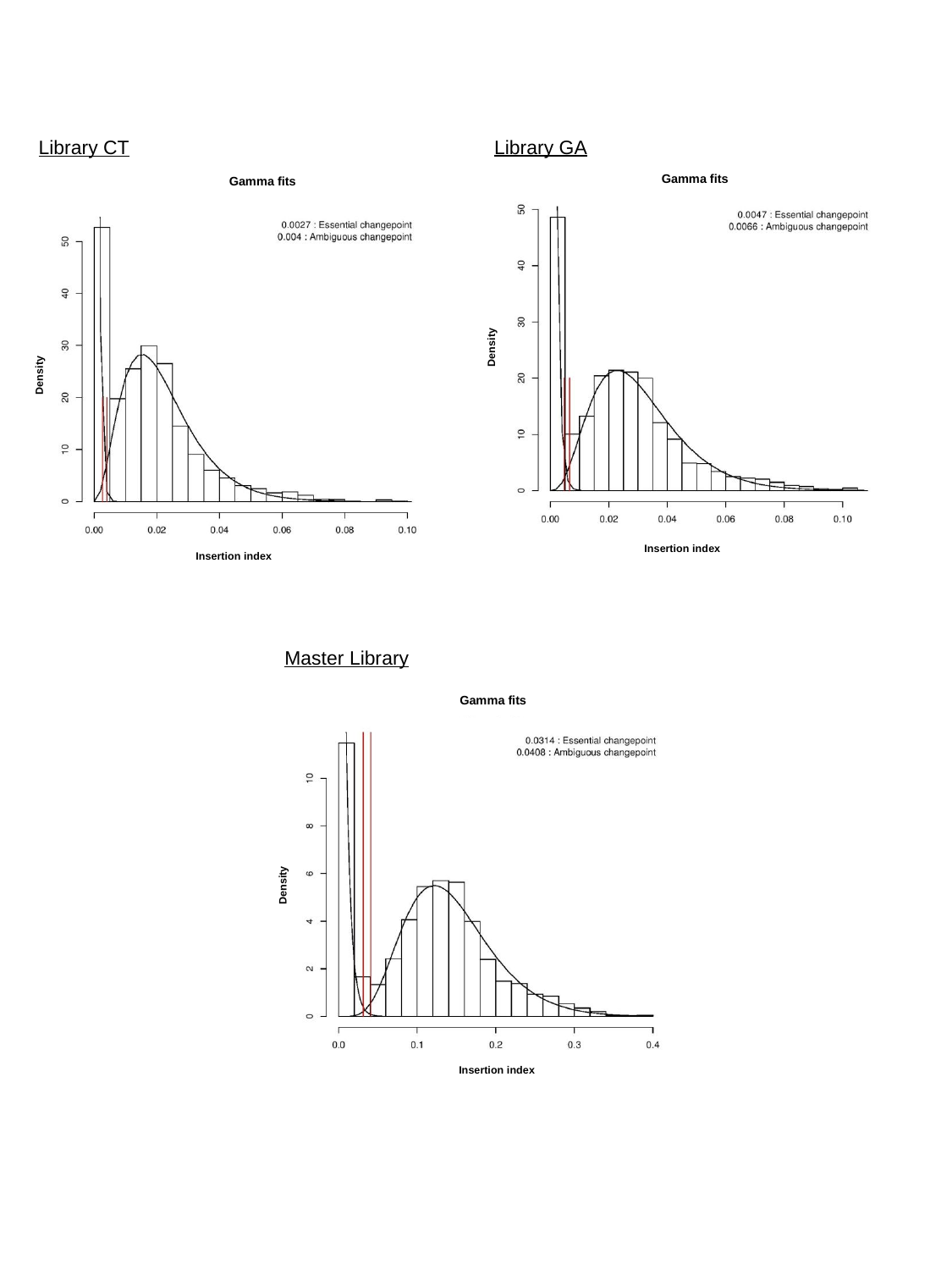

Library GA
Gamma fits
Density
Insertion index
Library CT
Gamma fits
Density
Insertion index
Master Library
Gamma fits
Density
Insertion index
